# A Comparison of the Corrected Intraocular Pressure Obtained by the Corvis ST and Reichert 7CR Tonometers in Glaucoma Patients

**DOI:** 10.1371/journal.pone.0170206

**Published:** 2017-01-17

**Authors:** Yoshitaka Nakao, Yoshiaki Kiuchi, Satoshi Okimoto

**Affiliations:** Ophthalmology and Visual Science Department, Hiroshima University, Hiroshima, Japan; Bascom Palmer Eye Institute, UNITED STATES

## Abstract

The purpose of the study was to investigate the accuracy of two corrected intraocular pressure (IOP) measurements by Corvis Scheimpflug Technology (CST)-IOPpachy and by corneal-compensated IOP (IOPcc) using the Reichert 7CR (7CR) tonometers. We also investigated the effects of corneal anatomical and structural parameters on the IOP measurements. The participants included 90 primary open-angle glaucoma patients. We assessed the IOP measurements, obtained by the CST, 7CR, and Goldmann applanation tonometer (GAT), using a paired *t*-test with Bonferroni correction, Bland-Altman plots, and multiple regression analyses. The 7CR-IOPcc gave the highest value (15.5 ± 2.7 mmHg), followed by the 7CR-IOPg (13.7 ± 3.1 mmHg), GAT-IOP (13.6 ± 2.2 mmHg), CST-IOP (10.3 ± 2.6 mmHg), and CST-IOPpachy (9.7 ± 2.5 mmHg). The values of CST-IOPpachy were significantly lower than those obtained by the other IOP measurement methods (all, p < 0.01). The values of 7CR-IOPcc were significantly higher than those obtained by the other IOP measurement methods (all, p < 0.01). Bland-Altman plots showed a mean difference between the GAT-IOP and the other IOP measurements (CST-IOP, CST-IOPpachy, 7CR-IOPg, and 7CR-IOPcc), which were −3.20, −3.82, 0.14, and 2.00 mmHg, respectively. The widths of the 95% limits of agreement between all pairs of IOP measurements were greater than 3 mmHg. With the exception of the 7CR-IOPcc, all of the IOP variations were explained by regression coefficients involving gender, average corneal curvature, and central corneal thickness. The IOP values obtained by the GAT, CST, and 7CR were not interchangeable. Each new IOP measurement device that was corrected for ocular structure had its own limitations.

## Introduction

Intraocular pressure (IOP) is a fundamental parameter in every ophthalmic examination, and it is of critical importance in the management of patients with glaucoma. It is therefore important to accurately assess the IOP of glaucoma patients. The ideal tonometer should be accurate, and yield reproducible results that are minimally influenced by corneal properties. The Goldmann applanation tonometer (GAT) has several excellent characteristics, including high accuracy and high reproducibility [1, 2], and it is presently the clinical standard for IOP measurements. However, corneal biomechanical properties, including the central corneal thickness (CCT), influence IOP measurements when using this device [3–5].

More recently, numerous novel devices have been introduced to measure the IOP without being affected by ocular biomechanical parameters, such as CCT and aging. These include the Corvis Scheimpflug Technology (CST; Oculus, Wetzlar, Germany), the Ocular Response Analyzer (ORA; Reichert, Delpew, NY, USA), and the Reichert 7CR (7CR; Reichert). Table 1 lists a comparison of these instruments. These devices measure IOP based on corneal deformation information in response to an air impulse. In brief, they apply a precisely metered collimated air impulse to the corneal apex. The air impulse causes the cornea to move inwards. When the pressure of the air puff decreases, the cornea gradually returns to its normal configuration. The 7CR and ORA measure the intensity of the reflected infrared light from the deforming corneal surface. The signal intensity of the reflected infrared light is strongest when the applanated corneal surface is flat. In contrast, the Corvis-ST Scheimpflug imaging system scans a larger surface and is a more accurate determination of the corneal deformation process. This system records the deformation process with 4,330 Scheimpflug images per second and can detect Scheimpflug images of the applanation of the cornea. The 7CR and ORA evaluate the IOP based on the inward and outward applanation points. The IOP readings are calculated using the average of the inward and outward applanation powers, which is called the Goldmann-correlated IOP (IOPg) and is calibrated to correspond with the GAT-IOP values. The algorithm-modified difference between the inward and outward applanations is then used to further adjust the IOP measurements weakly associated with corneal viscoelastic properties and thickness; these values are referred to as the corneal corrected IOP (IOPcc) [6]. The IOP measurement using the CST is based only on the inward applanation point, and is called the CST-IOP; it does not consider the CCT. However, a recent software update for the CST used a correction formula [corrected IOP = measured IOP + k − age (550 − CCT)] to correct the CST-IOP, called CST-IOPpachy, based on the patient’s CCT and age. The corneal biomechanical properties should theoretically have less influence on the IOPcc and CST-IOPpachy readings.

**Table 1:** Product comparison.

	Corvis Scheimpflug Technology (CST)	Ocular Response Analyzer (ORA)Reichert 7CR (7CR)
What causes the applanation of the cornea?	Air impulse	Air impulse
What is used to detect the applanation of the cornea?	4,330 Scheimpflug images per second	The signal intensity of the reflected infrared light
What is used to calculate the IOP values?	Inward applanation points	Inward and outward applanation points
Non-corrected IOP	CST-IOP	Goldmann-correlated IOP (IOPg)
Corrected IOP	CST-IOPpachy	Corneal corrected IOP (IOPcc)
What factors are weakly associated with the corrected IOP?	Central corneal thickness and age	Corneal viscoelastic properties and thickness

IOP, intraocular pressure; CST, Corvis Scheimpflug Technology.

Unfortunately, the ORA device was not available in Japan during the study period, so we compared the IOP measurements obtained by the CST, 7CR, and GAT in glaucoma patients. Of particular interest were the differences between the IOPcc (corneal compensated IOP using the 7CR) and IOPpachy (CCT and the aging corrected IOP using the CST). The effects of varying anatomical structures on the IOP measurements obtained by the three tonometers were also assessed.

## Materials and Methods

This study was a prospective, comparative analyses of IOP measurements obtained from the right eye of primary open-angle glaucoma (POAG) patients. The Hiroshima University Hospital’s institutional review board approved the study. With IRB permission, waivers of consent for prospective chart review were obtained based on an explanation of the implications of such activities, listed on a poster in the waiting rooms. In addition, it was registered with the University Hospital Medical Network (UMIN) clinical trials registry. The registration title was “Intercomparison analysis of factors obtained from corneal biomechanical parameters measurements having different measuring principles” and the registration number was JPRN-UMIN000016623. All of the study methods adhered to the tenets of the Declaration of Helsinki. The Department of Ophthalmology of Hiroshima University Hospital recruited 90 participants with POAG, from September 2014 to March 2015.

The exclusion criteria included: corneal pathological conditions that could affect the measurement of the IOP; a refractive error ≤ −6.00 diopters (D) equivalent sphere, and corneal astigmatism ≥ 3.00 D; a history of refractive, corneal, or incisional glaucoma surgery; secondary glaucoma; any visual field loss due to non-glaucomatous pathology (including retinal, optic nerve, or visual pathway disorders); low quality Scheimpflug images (indicated in red or yellow) that could not be automatically analyzed; and 7CR measurements with a waveform score < 7.0.

All patients underwent a full ophthalmic examination that included measurement of the spherical equivalent refraction and the corneal curvature (KR-800^®^; Topcon Corporation, Tokyo, Japan), the CCT (CASIA^®^; TOMEY Corporation, Aichi, Japan), and the axial length of the eye (IOL master^®^; Carl Zeiss Meditec AG, Jena, Germany). The average corneal curvature was calculated as the average of the horizontal and vertical corneal curvatures. The instruments used for the IOP measurements were two newly developed noncontact tonometers (CST and 7CR) and a clinical standard tonometer (GAT). During a single visit, the IOP was measured three times with each device by an experienced clinicians between 10:00 and 17:00 hours, with the patient in the sitting position. In all cases, the IOP measurements using the 7CR and CST were obtained in a randomized order, by the same clinicians with a 5-minute interval separating each device. Following these measurements, the subject was instructed to sit down, and a clinician masked the 7CR and CST results. After approximately 30 minutes for data masking, topical anesthesia consisting of 0.4% oxybuprocaine hydrochloride and fluorescein staining was applied, and then the ophthalmologist measured the IOP using the GAT.

We compared the IOP measurements by five methods using multiple comparisons. The sample size estimates were based on the standard deviation of the differences between IOPs (2.5 mmHg); minimal clinically important difference (1.0 mmHg); significance level (0.05); and power (80%). The estimated sample size was therefore 88 eyes.

### Statistical analysis

The data were expressed as the mean ± standard deviation (SD). Inter-device differences were evaluated using the repeated measures ANOVA and a paired *t*-test with Bonferroni correction. The 95% limits of agreement (LOA) between methods (the mean difference ± 1.96 SD contained 95% of the inter-method differences) was evaluated by Bland-Altman plots, which also assessed simultaneous visual examinations for both fixed bias (any systematic difference between the measurements) and proportional bias (any relationship of the discrepancies between the measurements and the averaged IOP value). We used univariate regression models to study patient parameters (gender, age, spherical equivalent, average corneal curvature, axial length, and CCT) that might be associated with the IOP measurements obtained by each of the three tonometers. Subsequently, all subset and stepwise multivariate regression analyses were used to construct models that best identified the parameters that were independently associated with these IOP measurements. For the potential collinearity problem, we utilized the variance inflation factor (VIF). VIF showed the variance inflation factor for each term in the model. A VIF > 5.0 indicated a collinearity issue among the terms in the multivariate regression analyses. P values < 0.05 were considered to indicate statistical significance. The statistical analyses were performed using JMP^®^, version 11.2.1 (SAS Institute Inc., Cary, NC, USA) and R, version 2.8.1 software.

## Results

Table 2 summarizes the demographic data. The CST, 7CR, and GAT were used to measure the IOP of the right eyes of 99 participants. The CST, 7CR, and GAT successfully measured the IOP in 90 eyes. For the CST and 7CR measurements, high quality Scheimpflug images were obtained in 95 eyes of the participants (96.0%) and waveform scores ≥ 7.0 were obtained in 93 eyes of the participants (94.0%). Therefore, further analyses used 90 eyes of 90 participants [40 female and 50 male, all eyes on anti-glaucoma medication, including 71 eyes on F2a-prostaglandin (PG) analogues].

**Table 2:** Characteristics of primary open-angle glaucoma patients (n = 90).

Characteristic	Mean ± SD	Range
Age (year)	61.94 ± 11.35	30.8–82.0
Gender (female/male)	40/50	
Spherical equivalent (diopter)	-2.00 ± 1.66	-5.6–1.4
Average corneal curvature (mm)	7.72 ± 0.27	7.1–8.5
Axial length (mm)	25.22 ± 1.62	22.1–28.7
Central corneal thickness (μm)	524.43 ± 31.22	446–617

SD, standard deviation.

**Fig 1:**
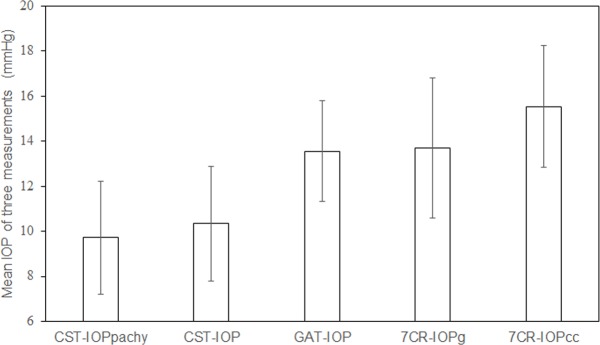
A comparison of mean IOP of three measurements using five methods. There were mean IOP of three measurements by each method on each patient (n = 90). The IOP values are plotted as the mean ± standard deviation. IOP, intraocular pressure; CST-IOP, IOP using the Corvis ST; CST-IOPpachy, corrected CST-IOP; GAT-IOP, IOP using the Goldmann applanation tonometer; 7CR-IOPg, Goldmann-correlated IOP using the 7CR tonometer; 7CR-IOPcc, corneal-compensated IOP using the 7CR tonometer.

Fig 1 shows a comparison of the mean IOP of three measurements obtained by the different devices. Overall, the 7CR-IOPcc measurements resulted in the highest values [15.5 ± 2.7 mmHg (mean ± SD)], followed by 7CR-IOPg (13.7 ± 3.1 mmHg), GAT-IOP (13.6 ± 2.2 mmHg), CST-IOP (10.3 ± 2.6 mmHg), and CST-IOPpachy (9.7 ± 2.5 mmHg). A repeated measures ANOVA showed that there was a significant difference between the IOP measurements (all, p <0.001). Using the paired *t*-test with Bonferroni correction, CST-IOPpachy showed significantly lower values than CST-IOP (p < 0.001), and both the CST-IOPpachy and CST-IOP values were significantly lower than the values from GAT-IOP, 7CR-IOPg, and 7CR-IOPcc (all, p < 0.001). The 7CR-IOPcc showed significantly higher values than the CST-IOPpachy, CST-IOP, GAT-IOP, and 7CR-IOPg (all, p < 0.001); however, there were no significant differences between the 7CR-IOPg and GAT-IOP (p = 0.52).

**Fig 2:**
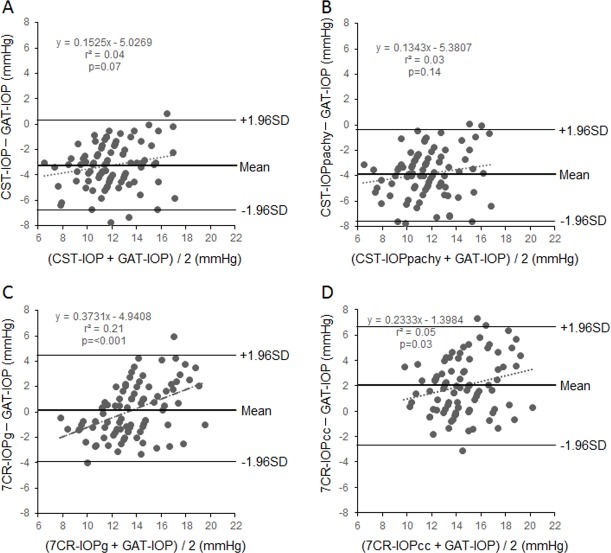
Bland-Altman plots for inter-method differences. A, The differences between the CST-IOP and GAT-IOP. B, The difference between the CST-IOPpachy and GAT-IOP. C, The difference between the 7CR-IOPg and GAT-IOP. D, The difference between the 7CR-IOPcc and GAT-IOP. The mean values and 95% LOA are indicated by bold lines and solid lines, respectively. IOP, intraocular pressure; CST-IOP, IOP using the Corvis ST; CST-IOPpachy, corrected CST-IOP; GAT-IOP, IOP using the Goldmann applanation tonometer; 7CR-IOPg, Goldmann-correlated IOP using the 7CR tonometer; 7CR-IOPcc, corneal-compensated IOP using the 7CR tonometer; CI, confidence interval; LOA, limits of agreement.

Fig 2 shows Bland-Altman plots showing the agreement between the IOP measurements. Fig 2A shows a comparison between the CST-IOP and GAT-IOP. The mean difference was −3.20 mmHg. The 95% LOA was the narrowest at 3.43 mmHg. A fixed bias was present, but we did not detect a proportional bias (r^2^ = 0.04; p = 0.07). Fig 2B shows the results of a comparison between the CST-IOPpachy and GAT-IOP. The mean difference was −3.82 mmHg, and the 95% LOA was 3.67 mmHg. We identified a fixed bias, but did not detect any proportional bias (r^2^ = 0.03; p = 0.14). Fig 2C shows a comparison between the 7CR-IOPg and GAT-IOP. The mean difference was 0.14 mmHg, and the 95% LOA was 4.05 mmHg. We did not detect a fixed bias, but we identified a weak proportional bias (r^2^ = 0.20; p < 0.001). Fig 2D shows a comparison between the 7CR-IOPcc and GAT-IOP. The mean difference was 2.00 mmHg. This comparison resulted in the widest 95% LOA (4.42 mmHg). In addition, we identified both a fixed and very weak proportional biases (r^2^ = 0.05; p = 0.03).

Using univariate regression analyses, gender, average corneal curvature, and CCT each were associated with the IOP in at least one of the IOP measurements (Table 3). We used stepwise multivariate regression analyses to adjust for the interactions between variables. We found that the average corneal curvature (β = −2.56; p = 0.009) independently influenced the CST-IOPpachy measurement, and the average corneal curvature (β = −2.67; p = 0.004) and CCT (β = 0.03; p < 0.001) independently influenced the CST-IOP measurement. Gender (β = 0.52; p = 0.03) influenced the GAT-IOP measurement, and CCT (β = 0.03; p = 0.005) influenced the 7CR-IOPg measurement. None of these factors influenced the 7CR-IOPcc measurement. We found that all the VIFs in identified factors in the stepwise multivariate regression analysis were 1.0.

**Table 3:** The results of univariate and multiple regression analyses: Factors independently associated with the intraocular pressure measurements (β, p, VIF).

	IOP measurements														
Independent variables	CST-IOPpachy			CST-IOP			GAT-IOP			7CR-IOPg			7CR-IOPcc		
	β	p	VIF	β	p	VIF	β	p	VIF	β	p	VIF	β	p	VIF
Univariate regression analysis															
Sex (female)	0.32	0.229		0.55	**0.045**		0.61	**0.010**		0.72	**0.029**		0.35	0.227	
Age (year)	-0.02	0.367		-0.02	0.482		-0.02	0.282		-0.02	0.454		0.00	0.980	
Spherical equivalent (diopter)	-0.01	0.935		0.00	0.989		-0.06	0.665		0.08	0.691		0.16	0.365	
Average corneal curvature (mm)	-2.34	**0.015**		-2.40	**0.015**		-1.32	0.129		-1.13	0.351		-0.11	0.920	
Axial length (mm)	-0.18	0.280		-0.18	0.285		-0.10	0.506		-0.17	0.404		-0.14	0.438	
Central corneal thickness (μm)	0.00	0.708		0.03	**0.002**		0.02	**0.025**		0.03	**0.001**		0.01	0.529	
Stepwise multivariate regression analysis															
Sex (female/male)							0.52	**0.027**	1.0	0.53	0.098	1.0			
Age (year)	-0.03	0.173	1.0												
Spherical equivalent (diopter)															
Average corneal curvature (mm)	-2.56	**0.009**	1.0	-2.67	**0.004**	1.0									
Axial length (mm)															
Central corneal thickness (μm)				0.03	**0.001**	1.0	0.01	0.071	1.0	0.03	**0.005**	1.0			

P values < 0.05 are shown in bold.

VIF, variance inflation factor (VIF > 5.0 indicates a collinearity issue); IOP, intraocular pressure; CST-IOP indicates the IOP using the Corvis ST; CST-IOPpachy, corrected CST-IOP; GAT-IOP, IOP using the Goldmann applanation tonometer; 7CR-IOPg, Goldmann-correlated IOP as measured by the 7CR tonometer; 7CR-IOPcc, corneal-compensated IOP as measured by the 7CR tonometer.

## Discussion

### Comparison of the five IOP measurements

The relationships between the IOP measurements were as follows. The CST-IOPpachy values were lower than the CST-IOP values. Both the CST-IOPpachy and CST-IOP values were significantly lower than the other IOP values. The GAT-IOP values were equal to the 7CR-IOPg values. The 7CR-IOPcc values were the highest of the five IOP readings.

Some previously reported CST-IOP measurements were higher than the GAT-IOP measurements [7–10] and other studies reported that the CST-IOP was similar to [11] or was lower than [12] the GAT-IOP. Previous comparisons between the CST-IOP and GAT-IOP were controversial [7–12]. The CST-IOPpachy was designed to reduce the effect of the CCT and aging. In our results, the CST-IOPpachy values (which consider CCT) were lower than the CST-IOP values, and univariate and stepwise multivariate regression analyses indicated that the CST-IOPpachy values were calibrated to eliminate the CCT effect. These results suggested to us that our patients had a thick cornea. However, their CCT (524 ± 31.2 μm) was similar to the average CCT (518 ± 29 μm) of Japanese POAG patients [13]. The exact reason for this difference between the CST-IOPpachy and the CST-IOP values is unknown.

In our study, there was no significant difference between the GAT-IOP and 7CR-IOPg measurements. We hypothesize that the IOPg was designed to give the closest IOP measurement, as was the GAT.

Vincent et al. [14]and Gungor et al. [15]reported that the 7CR-IOPcc values were approximately 2 mmHg higher than the GAT-IOP values. Our results are consistent with their previous results. The reason may be that glaucoma patients were treated with glaucoma medications, such as PG analogues, which induced a mild decrease in the CCT [16]. The GAT-IOP value could be influenced by the decreased CCT and be lower than the true IOP value in these patients.

Because the CST-IOPpachy was designed to reduce the effect of the CCT, the values would be expected to be higher than the CST-IOP and GAT-IOP values when assuming a PG analogue effect. However, the CST-IOPpachy values were lower than the CST-IOP or GAT-IOP values. The PG analogue effect on CCT therefore cannot explain the IOP reading differences among the tonometers. The exact reason for this discrepancy is unknown; however, differences in the detection ability of the tonometers in flat applanated conditions might account for this discrepancy.

### Agreement between the two corrected IOP measurements and the GAT-IOP

Many previous reports [9, 10, 14, 15, 17, 18] have compared the GAT-IOP with the IOPcc, but no reports have shown good agreement between these two parameters. In the present study, we compared the GAT-IOP and 7CR-IOPcc measurements, and the GAT-IOP and CST-IOPpachy measurements. We found that the mean difference between the two corrected IOP measurements (7CR-IOPcc and CST-IOPpachy) and the GAT-IOP were 2.00 and −3.82 mmHg, with 95% LOA widths of 4.42 and 3.67 mmHg, respectively. Fixed biases were identified between the two corrected IOP measurements and the GAT-IOP measurements. Proportional biases were identified between the 7CR-IOPcc and GAT-IOP measurements, but not between the CST-IOPpachy and GAT-IOP measurements. Based on these results, the CST-IOPpachy and 7CR-IOPcc devices were not interchangeable with respect to the GAT.

### The effects of anatomical and structural factors on IOP measurements

Many previous reports used regression analyses to investigate the relationships of IOP measurements using the CST and ORA to measure anatomical and structural parameters such as the CCT [7–9, 11, 12, 18, 19], corneal curvature [9, 11, 18, 19], spherical equivalent [9], and axial length [11, 18]. In the present study, we used stepwise multivariate regression analyses to comprehensively analyze the anatomical and structural factors that affected IOP measurements. We found that gender, the CCT, and the corneal curvature were significant factors. Gender (β = 0.52; p = 0.03) was associated with the GAT-IOP; the GAT-IOP of females was higher than that of males. We could not find studies that reported whether gender influenced the accuracy of IOP measurements. Ocular rigidity is a parameter that influences the ability to measure the true IOP [20]. The corneal structure and rigidity exhibit sexual dimorphism [21, 22]. Nakakura et al. [23] measured the amount of corneal displacement by photographing the corneal profile at 5,000 frames/s (1 frame/0.2 ms) with a high-speed camera during noncontact tonometry. They reported that the age-related reduction in corneal rigidity was greater in females than in males.

The CCT did not affect the CST-IOPpachy and 7CR-IOPcc values in this study, because these values were calibrated to eliminate the CCT effect. In contrast, the corneal curvature affected the CST-IOPpachy (β = −2.56; p = 0.009) and CST-IOP (β = −2.67; p = 0.004) values. Liu et al. [24] reported that a steeper curvature had a more significant impact on the accuracy of IOP measurements than a flatter curvature, and that IOP readings increased as the corneal curvature decreased. The corneal curvature did not affect the GAT-IOP (β = −1.32; p = 0.1293) readings. Mark [25] previously reported that the corneal curvature affected the GAT readings. However, Kohlhaas et al. [26] and Francis et al. [27] recently reported a lack of correlation between the IOP readings and the corneal curvature. Although the corneal curvature may affect the GAT-IOP readings, this effect would be marginal in comparison with the effect of the CCT.

Age-related changes of the collagen framework may affect ocular tonometry by changes in corneal biomechanical properties [28]. This possibility suggested to us that there was a significant positive correlation between age and IOP measurements. However, previous reports [12, 29, 30] and our results contradicted this hypothesis. Although the CST uses a correction formula to correct the CST-IOP based on the patient’s CCT and age, further studies are required to determine whether it is more useful to correct for the corneal curvature and the CCT.

Using stepwise multivariate regression analysis, there was no high VIF, indicating that there was no collinearity problem among the terms in the model.

### The reliability of the CST and 7CR measurements

In our study, the IOP was successfully measured using CST in 96.0% and using 7CR in 94.0% of the patients, respectively. These results suggested that each device had a high success rate measurement, but the success rate measurement using the CST was better than that of the 7CR. Several studies utilized an intraclass correlation coefficient (ICC) to evaluate the reliability of the CST-IOP and IOPcc measurements. They found that the ICC of the CST-IOP was very good [7, 11, 12, 31] and the ICC of the IOPcc was good to very good [32–34]. We suggest the reason was due to the presence or absence of an eye monitor during tonometry. In our experience, some patients were startled by the first of three measurements on the same eye. The patient’s eye was sometimes unable to fix on the fixation point, which caused alignment problems. Using the CST, the operator could obtain information about the patient’s condition by using an observation monitor. In addition, the monitor helped to align the center position between the center positioning mark and the center of the pupil. In contrast, the timing of the measurement and the position alignment were automatically controlled using the 7CR. There was no observation monitor, thus the operator could not obtain information about the patient’s condition. Automated timing of the measurement and position alignment system without an observation monitor can enhance usability and make IOP measurements easier for the operator, but this system sometimes reflects the potential disadvantages in reliability of the 7CR measurements.

### The advantages and disadvantages of the CST and 7CR

The CST device initially only evaluated the CST-IOP. Subsequently, an updated CST software program, the corrected IOP measurement, as CST-IOPpachy, based on the patient’s CCT and age was added. However, our results showed that the CST-IOPpachy still did not measure accurate levels of the IOP, because the CST-IOPpachy was affected by corneal biomechanical properties such as the corneal curvature. The CST device is constantly being improved, so a better device should soon be available. In contrast, the 7CR device measured the correct IOP, as IOPcc, based on the patient’s corneal viscoelastic properties and thickness. Thus, IOPcc could be useful in the prognosis after refractive surgery [35–37]. However, there was no observation monitor in the 7CR, so the operator could not obtain information about the patient’s condition, and the measurements were sometimes not reliable.

Our study involved some limitations. First, we only included glaucoma participants treated with glaucoma medications in our comparison of the three tonometers. These patients generally were treated with glaucoma medications, which induce mild changes in corneal structure and other properties [16]. Consequently, the corneal structure of glaucoma patients may differ from that of patients without treatment. This also suggests that our results may not be applicable to patients without glaucoma. Because glaucoma medications reduce elevated IOP, the IOP measured in our glaucoma patients were within the normal range, so our results may not be applicable in patients with a higher IOP. Second, Bolivar et al. [38] reported that treatment with PG analogues might have an effect on corneal hysteresis. Unfortunately, the 7CR tonometer does not consider corneal hysteresis, so we could not analyze the relationship between corneal hysteresis and IOP measurements. Third, we evaluated the CST and 7CR devices in comparison with the GAT, using GAT as a clinical standard. However, the GAT-IOP does not provide the correct IOP, and it is still not known which IOP measurement is closest to the true value.

## Conclusions

Measurements of the IOP by the CST-IOPpachy were lower than the GAT-IOP measurements, and those of the 7CR-IOPcc were consistently higher than those of the GAT-IOP. The IOP values obtained by these three tonometers were not interchangeable. CST-IOPpachy measurements were affected by the corneal curvature, and the CCT and corneal curvature did not affect the 7CR-IOPcc readings. IOP measurements using the 7CR-IOPcc or CST-IOPpachy corrected for ocular structures, but were associated with their own limitations.

## Supporting Information

S1 Table All relevant data. The data of the right eyes of 90 participants.
(XLSX)
