# Correction: A Comparison of the Corrected Intraocular Pressure Obtained by the Corvis ST and Reichert 7CR Tonometers in Glaucoma Patients

**DOI:** 10.1371/journal.pone.0173306

**Published:** 2017-02-28

**Authors:** The PLOS ONE Staff

Due to issues with the typesetting process, there are errors in Table 3. The publisher apologizes for the errors. Please see the correct Table 3 here.

**Table 3:** The results of univariate and multiple regression analyses: Factors independently associated with the intraocular pressure measurements (β, p, VIF).

Independent variables	IOP measurements														
	CST-IOPpachy			CST-IOP			GAT-IOP			7CR-IOPg			7CR-IOPcc		
	β	p	VIF	β	p	VIF	β	p	VIF	β	p	VIF	β	p	VIF
Univariate regression analysis															
Gender (female)	0.32	0.229		0.55	**0.045**		0.61	**0.010**		0.72	**0.029**		0.35	0.227	
Age (year)	-0.02	0.367		-0.02	0.482		-0.02	0.282		-0.02	0.454		0.00	0.980	
Spherical equivalent (diopter)	-0.01	0.935		0.00	0.989		-0.06	0.665		0.08	0.691		0.16	0.365	
Average corneal curvature (mm)	-2.34	**0.015**		-2.40	**0.015**		-1.32	0.129		-1.13	0.351		-0.11	0.920	
Axial length (mm)	-0.18	0.280		-0.18	0.285		-0.10	0.506		-0.17	0.404		-0.14	0.438	
Central corneal thickness (μm)	0.00	0.708		0.03	**0.002**		0.02	**0.025**		0.03	**0.001**		0.01	0.529	
Stepwise multivariate regression analysis															
Gender (female)							0.52	**0.027**	1.0	0.53	0.098	1.0			
Age (year)	-0.03	0.173	1.0												
Spherical equivalent (diopter)															
Average corneal curvature (mm)	-2.56	**0.009**	1.0	-2.67	**0.004**	1.0									
Axial length (mm)															
Central corneal thickness (μm)				0.03	**0.001**	1.0	0.01	0.071	1.0	0.03	**0.005**	1.0			

P values < 0.05 are shown in bold.

VIF, variance inflation factor (VIF > 5.0 indicates a collinearity issue); IOP, intraocular pressure; CST-IOP indicates the IOP using the Corvis ST; CST-IOPpachy, corrected CST-IOP; GAT-IOP, IOP using the Goldmann applanation tonometer; 7CR-IOPg, Goldmann-correlated IOP as measured by the 7CR tonometer; 7CR-IOPcc, corneal-compensated IOP as measured by the 7CR tonometer.

## References

[1] Nakao Y, Kiuchi Y, Okimoto S (2017) A Comparison of the Corrected Intraocular Pressure Obtained by the Corvis ST and Reichert 7CR Tonometers in Glaucoma Patients. PLoS ONE 12(1): e0170206 doi:10.1371/journal.pone.0170206 28095506 10.1371/journal.pone.0170206 PMC5240967

